# Drug-Induced Oral Erythema Multiforme: A Report of a Rare Case

**DOI:** 10.7759/cureus.70977

**Published:** 2024-10-07

**Authors:** Shyamkumar Sriram, Mambakkam J Jayakanth, Sarah Mariam, Shazina Saeed, Shamimul Hasan

**Affiliations:** 1 Department of Rehabilitation and Health Services, College of Health and Public Service, University of North Texas, Denton, USA; 2 Department of Internal Medicine, Patiala Heart Institute, Patiala, IND; 3 Department of Periodontology, Bharati Vidyapeeth (Deemed to be University) Dental College and Hospital, Pune, IND; 4 Department of Epidemiology and Public Health, Amity Institute of Public Health & Hospital Administration, Amity University, Noida, IND; 5 Department of Oral Medicine and Radiology, Faculty of Dentistry, Jamia Millia Islamia, New Delhi, IND

**Keywords:** adverse drug reaction, lips, oral erythema multiforme, target lesions, vesiculobullous disorders

## Abstract

Erythema multiforme (EM) is an acute-onset, self-limiting inflammatory condition affecting the skin and mucous membranes. It exhibits a range of skin lesions, which is why it is referred to as "multiforme." Oral lesions, usually inflammatory and frequently marked by rapidly rupturing vesicles and bullae, often constitute a significant clinical feature. The exact etiology is obscure; however, it may be caused by multiple triggering factors. The most well-established connection is with a previous herpes simplex virus infection, while a smaller percentage of cases (<10%) are linked to drug use. Based on mucosal involvement, the condition is classified into two types: EM minor and EM major. Stevens-Johnson syndrome and toxic epidermal necrolysis (Lyell’s disease) are now recognized as separate clinical conditions. EM usually exhibits a self-limiting course, with lesions typically improving within a few weeks. Avoiding triggers and using steroid therapy can be effective treatments.

This paper documents a rare case of drug-induced oral EM featuring characteristic lip and intraoral lesions. The patient developed painful oral ulcers and difficulty in swallowing after taking azithromycin for a sore throat and fever. Clinical examination revealed multiple, hemorrhagic encrustations on the lips, along with ulcers on the right buccal mucosa and vesicular eruptions on the palate. The acute onset of multiple oral ulcers associated with a recent drug intake led to a diagnosis of drug-induced oral EM. Treatment included systemic prednisolone, topical triamcinolone paste, and supportive oral care. No recurrence was observed during the six-month follow-up.

## Introduction

Erythema multiforme (EM) is an acute, self-limiting, vesiculobullous condition that affects the skin, mucous membranes, or both, and often recurs [1]. It is characterized by distinctive target-shaped lesions with concentric color variation, usually appearing on the extremities, and may be accompanied by erosions of the oral, ocular, or genital mucosa, or a combination [2].

EM usually affects healthy young adults, with the highest incidence between ages 20 and 40, but around 20% of cases can also occur in children [3,4].

The exact etiopathogenesis of EM remains unclear, but it is thought to be an immune-mediated condition. While a considerable number of EM cases are idiopathic, they can occur after infections (such as herpes simplex virus and *Mycoplasma pneumoniae*), exposure to medications (like antibiotics), vaccinations, and autoimmune diseases. The clinical manifestations of EM result from the activation of cytotoxic T lymphocytes in the epithelium, leading to apoptosis in keratinocytes and subsequent necrosis of satellite cells [5].

EM is divided primarily into minor and major forms, based on the mucous membrane involvement. It may affect only the mouth or be accompanied by a skin eruption, with or without lesions involving the oral or other mucous membranes. EM minor generally affects only one mucosa and may be associated with symmetrical target-like skin lesions on the extremities. In contrast, EM major usually affects two or more mucous membranes and presents with a broader spectrum of skin involvement [6,7].

EM was once regarded as part of a spectrum of disorders encompassing Stevens-Johnson syndrome (SJS) and toxic epidermal necrolysis (TEN). However, EM is now acknowledged as a separate entity. It differs from SJS/TEN in etiology, clinical and histopathologic features, management, and prognosis [2,3,8]. It is essential to emphasize that EM is defined by papules, as it may be confused with SJS or TEN, which features macular lesions and a greater likelihood of mucosal involvement [9].

Oral lesions tend to affect the lips, buccal mucosa, and tongue, often appearing as erythematous macules and blood-tinged crusted lesions on the lips. Isolated oral lesions are uncommon and can be challenging to diagnose. This atypical form is known as oral EM [10].

The management of EM remains controversial due to insufficient conclusive evidence. Avoiding or managing potential triggers is crucial, and corticosteroids may be needed in severe cases [7].

This report describes a case of drug-induced oral EM to underscore the significant association between EM and medication use. It also includes a brief review of the etiology, clinical features, differential diagnosis, and treatment options for this condition.

## Case presentation

A 21-year-old male patient was referred by a general physician to our outpatient department for evaluation of painful oral ulcers and difficulty in swallowing for the past two days. History revealed that the patient consulted a general physician at the Internal Medicine Department, Patiala Heart Institute, Patiala for sore throat and fever five days back and was prescribed a three-day course of azithromycin (tablet Azithral 500 mg once daily). Within a day of starting the medication, he developed multiple vesicles on the lips and oral cavity, which later ruptured, forming irregular ulcers. The patient was advised topical application of Metrohex gel (0.25% chlorhexidine gluconate and 1% metronidazole), and oral antihistamines (levocetirizine 5 mg once daily); however, these treatments did not alleviate the symptoms. The patient was referred to the Oral Medicine Outpatient Department, Faculty of Dentistry, Jamia Millia Islamia, New Delhi for management of oral lesions.

**Figure 1:**
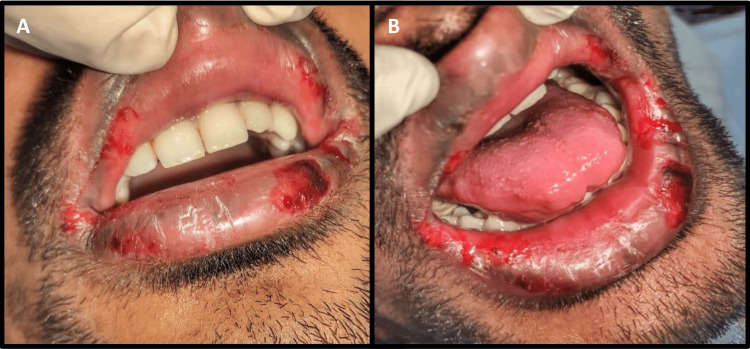
Clinical picture of the patient. The clinical picture shows multiple ulcerations with hemorrhagic encrustations on the upper and lower lips (A and B).

The general physical examination was unremarkable, showing no signs of systemic or nodal involvement, and all the vitals were within a normal range. On extra-oral examination, diffuse areas of multiple ulcerations with irregular borders and hemorrhagic encrustations were observed on the mucosa and vermillion border of both the upper and lower lips. The lips were slightly swollen and dry and showed fissures and cracks. On palpation, the ulcers were tender, and even gentle pressure resulted in bleeding (Figure 1).

**Figure 2:**
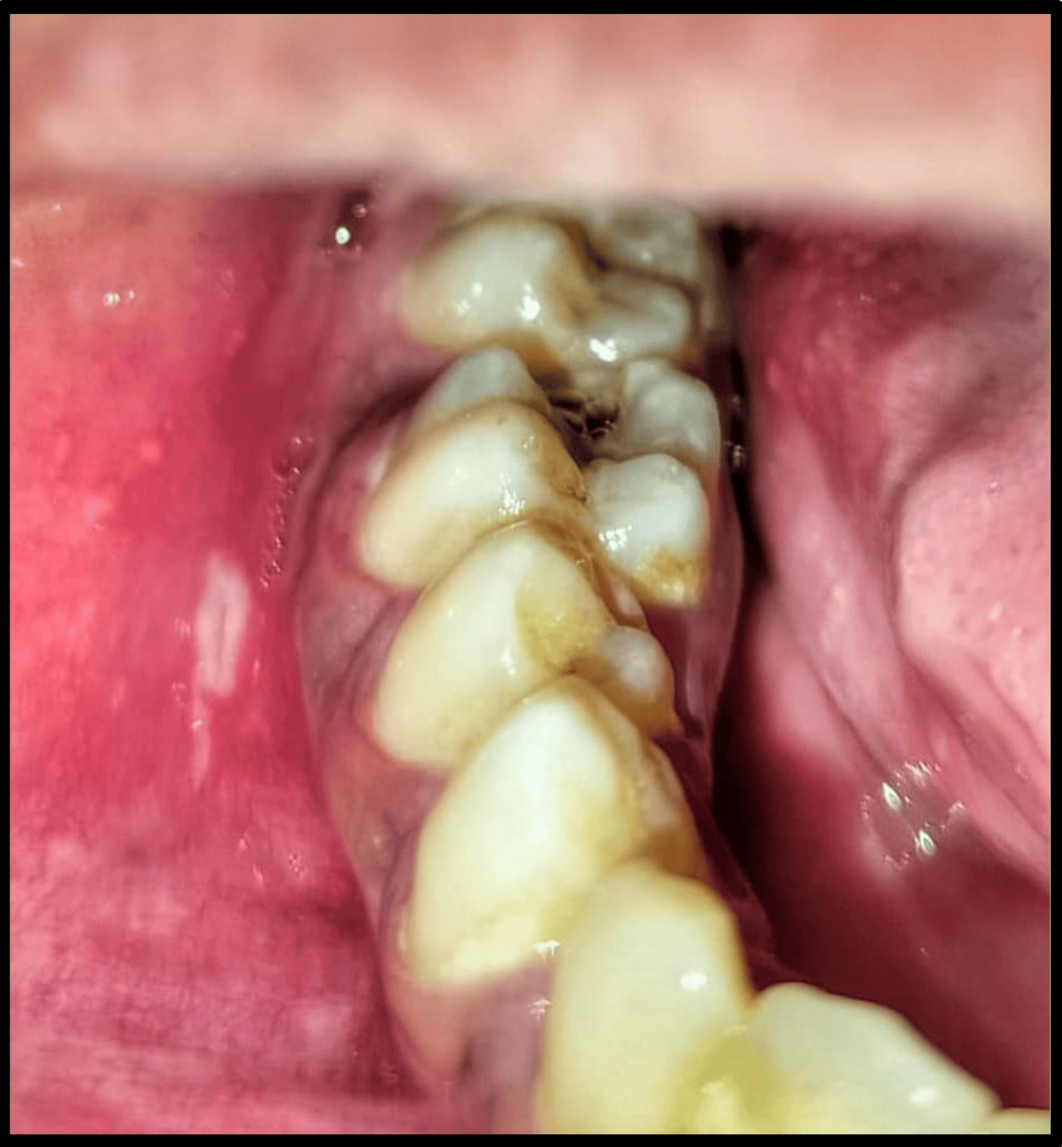
Intraoral clinical picture. Intraoral picture showing a large, elliptical ulcer on the right buccal mucosa. Multiple small, erythematous ulcers adjacent to the larger ulcer are also appreciable.

Intraoral examination revealed a large, elliptical ulcer on the right buccal mucosa adjacent to the mandibular posterior teeth. The ulcer measured about 0.8 x 0.6 cm, was surrounded by erythema, and had a yellowish pseudomembranous slough on its surface. Additionally, multiple small, erythematous ulcers were also observed on the right buccal mucosa adjacent to the larger lesion (Figure 2).

**Figure 3:**
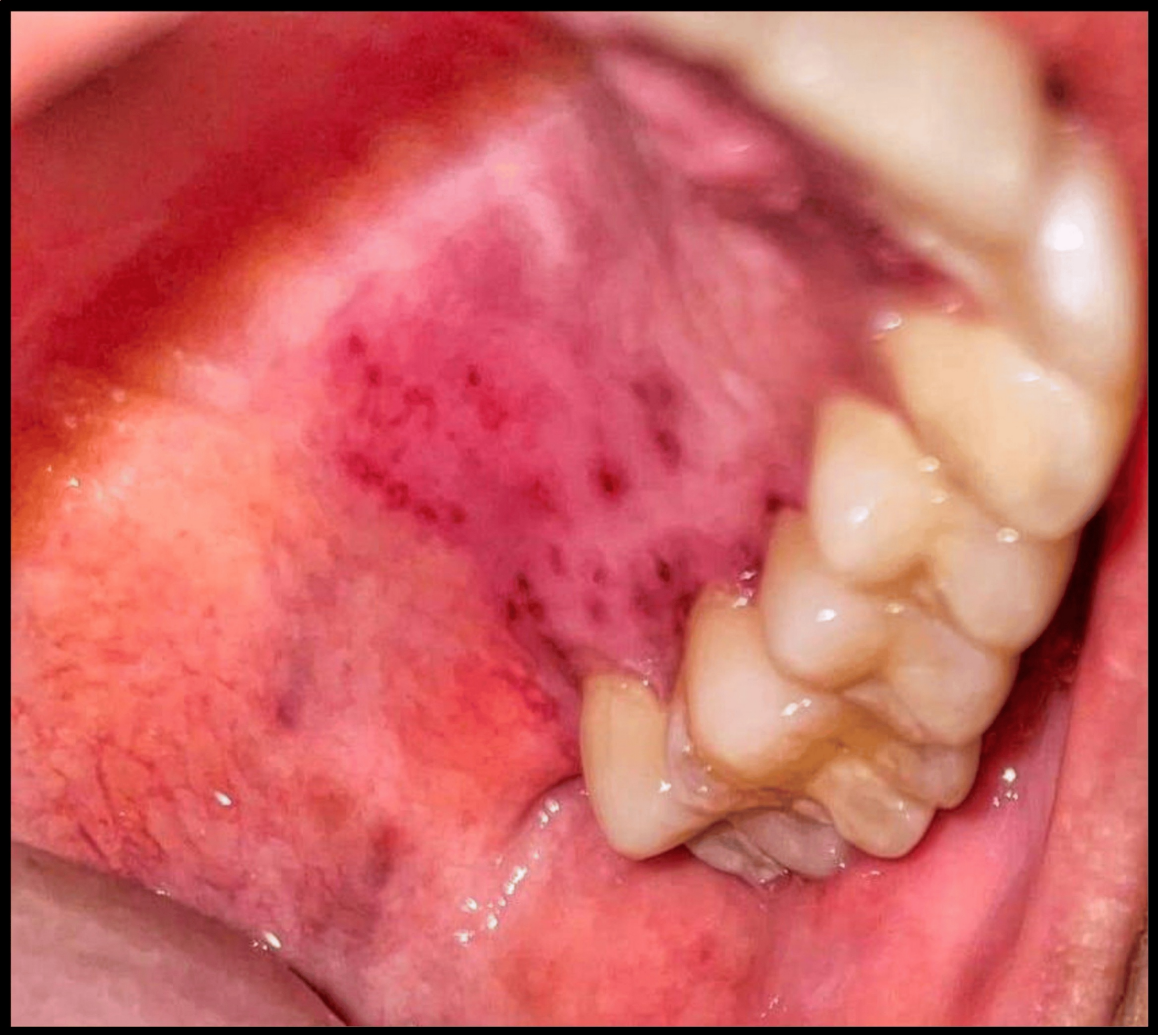
Intraoral clinical picture. Intraoral clinical picture reveals multiple, pin-point vesicular lesions on the hard palate.

Multiple pinpoint vesicular eruptions of different sizes, bordered by erythematous margins, were also noted on the left palatal mucosa adjacent to the left maxillary posterior teeth (Figure 3).

The patient experienced considerable discomfort, and the ulcers interfered with speech and swallowing. There were no associated lesions on the skin, genitals, or eyes, and Nikolsky’s sign was absent.

The sudden onset of multiple blood-tinged lip encrustations, coupled with a recent history of drug intake and the exclusion of other ulcerative lesions, strongly pointed to a diagnosis of drug-induced oral EM. Herpetic gingivostomatitis, bullous autoimmune disorders (such as pemphigus vulgaris, bullous pemphigoid, mucous membrane pemphigoid, bullous lichen planus, and paraneoplastic pemphigus), and adverse drug reactions, were considered in the differential diagnosis.

Routine hematological investigations were within the normal range. Molecular tests (polymerase chain reaction, PCR) and serological tests (enzyme-linked immunosorbent assay, ELISA) were performed to exclude any association with herpes simplex virus (HSV). PCR for HSV-1 and HSV-2 from the lip swab was negative. ELISA revealed non-reactive serum IgG and IgM for HSV.

The following hematological, molecular, and serological tests were conducted (Table 1).

**Table 1:** Various hematological, molecular, and serological investigations performed. Hb: hemoglobin; RBC: red blood cells; RCDW: red cell distribution width; MCV: mean corpuscular volume; MCH: mean corpuscular hemoglobin; MCHC: mean corpuscular hemoglobin concentrate; PCV: packed cell volume; WBC: white blood cells; ESR: erythrocyte sedimentation rate; g/dL: grams per deciliter; fL: femtoliter; pg/cell: picograms per cell; PCR: polymerase chain reaction; HSV: herpes simplex virus; ELISA: enzyme-linked immunosorbent assays.

Serial No.	Investigations	Reference range	Test results
Complete blood count (CBC)			
1.	Hb	Males - 13 to 18 g/dL; females - 12 to g/dL	14.6 g/dL
2.	RBC count	Males - 4.6 to 6.2 million cells/μL; females - 4.2 to 5.4 million cells/μL	4.8 million cells/μL
3.	RCDW	11.5% to 15%	12.2%
4.	MCV	80 to 100 fL	90.50 fL
5.	MCH	27-32 pg/cell	28.8 pg/cell
6.	MCHC	32-34.5 g/dL	32.8 g/dL
7.	PCV/hematocrit	Males - 40% to 54%; females - 36-48%	44%
8.	Total WBC count	4500 to 11000 cells/μL	8000 cells/μL
9.	Neutrophils	40-70%	64%
10.	Lymphocytes	20-40%	22%
11.	Monocytes	2-8%	3%
12.	Eosinophils	0-4%	1%
13.	Basophils	0-1%	0%
14.	Platelet count	150,000 to 400,000/μL	300,000/μL
15.	ESR (30 minutes)	0-8 mm	4 mm
16.	ESR (60 minutes)	05-15 mm	8 mm
Molecular tests			
17.	PCR (confirm the presence of HSV DNA even when antibodies are not detectable)	-	Negative
Serological tests			
18.	ELISA (for effective detection of antigens (HSV glycoproteins) or antibodies specific to HSV glycoproteins)	-	Non-reactive serum IgG and IgM for HSV

The patient was instructed to discontinue the medication and was prescribed systemic prednisolone (Wysolone 20 mg tablets, twice daily), topical application of Turbocort oromucosal paste (triamcinolone acetonide, 0.1%) three times a day, and tablet acyclovir 400 mg three times daily for five days. The antiviral therapy was discontinued after three days as the PCR and ELISA test results were negative for HSV. The patient was also instructed to perform oral rinses with Coolora mouthwash (benzydamine hydrochloride BP 0.15% w/v), maintain adequate fluid intake, and apply Vaseline to the lips to prevent them from sticking.

**Figure 4:**
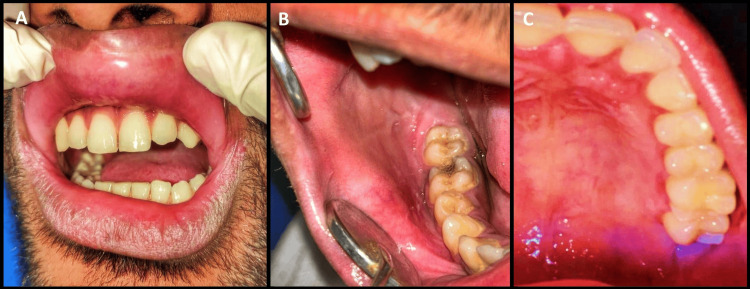
Post-treatment pictures. Healed lesions on the lips, buccal mucosa, and palate (A-C).

The lesions showed considerable regression within a week of steroid treatment. Systemic steroids were gradually tapered, and the patient experienced complete resolution of the lesions within 15 days (Figure 4).

During the six-month follow-up, there was no recurrence of the lesions.

## Discussion

EM is a type IV hypersensitivity reaction that predominantly targets keratinocytes, resulting in distinct ulcerative skin eruptions. The eruptions manifest as concentric erythematous rings, often called target lesions, and may also affect the oral cavity or other mucous membranes [6,11]. The name "multiforme" signifies the condition's varied clinical presentations, which include macules, papules, vesicles, and ulcers [1,5,12].

EM that appears solely in the oral cavity is uncommon. This form, called oral EM, presents with lesions on the oral mucosa and lips, without involvement of other body areas [10,11].

The exact etiopathogenesis of EM remains unclear; however, it is thought to be an immune-mediated reaction induced by an infectious agent or medication [4,13].

Approximately 90% of EM cases are associated with infections, with herpes simplex virus (HSV 1 & 2) being the main cause in adults and *Mycoplasma pneumoniae* in children [2,4,12,14]. Other microorganisms associated with EM include bacterial infections like mycobacteria and brucellosis; viral infections such as AIDS, adenovirus, enterovirus, and hepatitis viruses; and fungal infections including coccidioidomycosis, dermatophytosis, and histoplasmosis [2-8]. EM has also been reported with severe acute respiratory syndrome coronavirus 2 (SARS-CoV-2), the virus causing coronavirus disease 2019 (COVID-19) [5,15].

Drug-associated EM comprises less than 10% of cases. Commonly implicated drugs include antibiotics (such as sulphonamides, cephalosporins, penicillin, and ciprofloxacin), non-steroidal anti-inflammatory drugs (NSAIDs), anticonvulsants (like carbamazepine and phenytoin), cancer chemotherapeutic agents, allopurinol, and protease inhibitors [2,4,14,16,17].

In herpes-associated EM, HSV-DNA fragments in the skin or mucosa are likely to trigger the disease. CD34+ cells transport these HSV fragments to the epithelium, leading to the accumulation of T cells that respond to HSV antigens and cause cell damage. Conversely, drug-induced EM is thought to arise from reactive drug metabolites. Here, keratinocyte apoptosis is triggered by tumor necrosis factor-alpha released from keratinocytes, macrophages, and monocytes, leading to tissue damage [8,12,17].

Other known precipitants include chemicals or food additives (such as perfumes, benzoates, and nitrobenzene), malignancy, autoimmune conditions (like systemic lupus erythematosus (SLE), sarcoidosis, graft-versus-host disease, Bacillus Calmette-Guérin (BCG) vaccination, and inflammatory bowel disease), pregnancy, radiotherapy, menstruation, and psychological stress [2,16-18].

EM is most frequently observed in children and young adults (ages 20-40) and is uncommon in individuals over 50 years. It often presents with a sudden onset and severe cases may be accompanied by constitutional symptoms such as fever, malaise, and headache. EM may manifest in different forms, ranging from a mild, self-limiting exanthematous type with minimal oral involvement (EM minor) to a severe, rapidly progressing form marked by extensive mucocutaneous epithelial necrosis, known as SJS [4,5,17].

EM is categorized into major and minor forms according to the disease's severity and the locations of the lesions [2,4,5,8,13,16,17]. EM major is marked by ulceration across multiple mucosal sites and target-like lesions covering less than 10% of the body surface area (BSA). In contrast, EM minor, the least severe form, presents with target-like lesions involving less than 10% of the BSA and usually affects only one mucosal site, typically the mouth [3,4,11,16]. HSV-1 is the primary cause of EM minor, while EM major is more often linked to *Mycoplasma pneumoniae* infection [9].

Mucosal involvement usually coincides with skin lesions, though it can occur a few days before or after the appearance of skin lesions. In rare instances, patients may develop mucosal lesions without any associated skin lesions [2].

Oral EM is a distinct yet less frequently recognized variant of the condition. Initial episodes of oral EM usually affect only the oral mucosa and do not involve the skin. However, subsequent episodes may progress to a more severe form of EM that also involves the skin [3,10,12,19,20].

Oral lesions may be seen in 25% to 70% of cases of EM [2,4,8,12]. Oral lesions tend to affect the non-keratinized mucosa and the anterior regions of the oral cavity. The major affected regions include the lips (36%), followed by buccal mucosa (31%), tongue (22%), and labial mucosa (19%) [3,4]. The lips are often swollen and cracked and develop characteristic hemorrhagic crusts. Intact vesicles are seldom observed, as they tend to break down rapidly, resulting in ill-defined ulcers [4,13,16,17]. Oral lesions are marked by large, ill-defined erythematous ulcers, which may be accompanied by pseudomembranes or nonspecific hyperkeratotic plaques, along with bullae or erosions from ruptured blisters. However, the diverse clinical manifestations of EM may pose a diagnostic dilemma [2-4,13,17].

The cutaneous lesions initially present as discoid, erythematous macules that quickly evolve into papules. These papules then become edematous and develop into the targetoid or iris or bull's eye lesions typical of EM. The lesions generally appear symmetrically on the extensor surfaces of the limbs and can advance centrally, often involving both mucosal and skin areas [2-4,8,12,13].

"Typical targets" are characterized as individual lesions that are less than 3 cm in diameter, with a regular round shape, well-defined borders, and two concentric, edematous rings that appear lighter than the central disc. On the other hand, atypical lesions appear as raised, edematous areas with two distinct color zones and an ill-defined border [5-8,13,16,17].

The distribution pattern varies depending on its etiology: EM related to infections typically affects the extremities, while drug-induced EM generally involves the face and trunk. This distinction is crucial for determining the underlying cause of the disease [21].

EM is primarily a clinical diagnosis, relying on patient history and clinical manifestations, as histopathological and laboratory findings are often nonspecific [2-4,10,11,16-18]. Essential elements of the patient history include (a) an acute, self-limiting, or recurrent pattern; (b) features of associated infections, such as HSV or *M. pneumoniae*; and (c) recent drug intake. Clinical signs that aid in diagnosis include targetoid lesions, raised atypical papules, mucosal involvement, or any combination of these characteristics. Thus, combining the patient’s history with distinctive skin lesions and mucosal involvement can aid in confirming the diagnosis of EM [2,11].

The diagnosis of drug-induced EM relies on its distinctive clinical features and lesion distribution, a positive history of drug use linked to the abrupt onset of ulceration, and the exclusion of other infectious or inflammatory vesiculobullous conditions and drug reaction patterns [3,13,18,19].

Herpetic infections, autoimmune vesiculobullous disorders (including pemphigus vulgaris, bullous pemphigoid, mucous membrane pemphigoid, bullous lichen planus, and paraneoplastic pemphigus), as well as adverse drug reactions (such as fixed drug eruptions, lichenoid drug reaction, and anaphylactic stomatitis) should be included in the differential diagnosis of oral EM [3,10,13,16,19].

Acute herpetic gingivostomatitis presents with a viral prodrome, followed by the development of tiny blisters that quickly rupture, resulting in ulcerative lesions with an erythematous halo. It is commonly associated with marginal gingivitis and lymphadenopathy [22]. Also, herpetic ulcers are smaller and have more defined borders than those seen in EM [20].

Pemphigus vulgaris is identified by its chronic progression, the presence of skin lesions, flaccid intraepithelial vesicles or bullae, and a positive Nikolsky sign [23]. Also, the cutaneous lesions are bullous in PV in contrast to maculopapular lesions of EM [4].

Mucous membrane pemphigoid is a sub-epithelial blistering disorder that typically affects the mucous membranes (oral, genital, and conjunctival), with infrequent cutaneous lesions. Hemorrhagic blisters are usually short-lived, gingiva is the most frequently affected intraoral site (desquamative gingivitis), and healing with scarring is characteristically seen [24].

Bullous pemphigoid is typified by taut bullae, primarily affecting normal or erythematous skin with intense pruritus and symmetric lesions. Oral lesions are rarely observed, and Nikolsky’s sign is usually negative. Histologically, subepidermal blisters with inflammation are the hallmark features [25].

Erosive lichen planus is characterized by bilateral symmetrical reticular oral lesions. A lattice-like arrangement of white striations referred to as Wickham's striae covers the lesions and is most clearly observed in the buccal mucosa [26].

Severe cases of EM with hemorrhagic lip lesions can resemble paraneoplastic pemphigus. However, lesions in paraneoplastic pemphigus tend to be chronic, often present with severe ocular and skin involvement, and may carry a risk of malignancy [4].

Fixed drug eruption (FDE) is a drug-induced skin reaction that typically reappears in the same locations as prior episodes upon re-exposure to the drug. In our case, however, the lesions were widespread, involving the lips, buccal mucosa, and palate [12,13,16,20].

Treatment for EM varies based on the etiology and severity. Minor cases are often managed with supportive measures such as topical corticosteroids or oral antihistamines, while more severe cases necessitate systemic steroids. Hospitalization is needed only if mucosal lesions impede oral intake [2-5,8,13,16,18,20].

Initial treatment for EM minor focuses on managing infections and includes antiviral therapy for herpes-associated cases and antibiotics for *M. pneumoniae* cases [3,12,16,17]. In suspected drug-induced cases, promptly identifying and discontinuing the offending medication is essential to prevent the worsening of immune reactions. Additionally, it is crucial to avoid re-exposure to the same medication or to other drugs with similar chemical structures that could cause cross-reactivity [3,13,16,18,20].

Oral corticosteroids form the mainstay of treatment. Methylprednisolone is usually started at a minimum dose of 20 mg per day and adjusted to a maximum of 60 mg per day if needed. The dosage should then be gradually tapered over the next two to four weeks [2,12,16-18]. Dapsone, cyclophosphamide, azathioprine, levamisole, cyclosporine, thalidomide, and interferon-α are reserved for resistant cases [12,16].

## Conclusions

Drug-induced oral EM is a rare and under-reported variant of the condition. Although HSV infections are a common trigger for oral EM, drug reactions are less frequently involved. This case highlights the necessity of contemplating medication adverse effects in the diagnosis of acute mucosal ulcerations, as discontinuing the offending drug is pivotal for effective treatment. A meticulous patient history and thorough clinical examination are vital for an accurate diagnosis and appropriate treatment protocol.
